# Coaching presence as the foundation for the working alliance in AI coaching

**DOI:** 10.1371/journal.pone.0344768

**Published:** 2026-03-26

**Authors:** Gy Yoong Nam, Jinhee Choi

**Affiliations:** 1 Seoul Business School, aSSIST University, Seoul, Republic of Korea; 2 SDG Management School, Geneva, Switzerland; 3 Assistant Professor, Seoul Business School, aSSIST University, Seoul, Republic of Korea; Wroclaw University of Economics and Business: Uniwersytet Ekonomiczny we Wroclawiu, POLAND

## Abstract

Although Human Resources managers have integrated artificial intelligence (AI)-mediated coaching into their practice, the role of traditional coaching elements—especially coaching presence—within these interactions remains underexplored. To examine perceived differences in the working alliance in AI-mediated coaching, we conducted pre- and post-interviews with 15 Human Resources leaders from South Korean IT companies. Participants were categorized into three groups: Novice (no prior coaching experience), coaching-educated (completed formal coaching education without certification), and certified (completed both education and formal certification). Using reflexive thematic analysis, we explored how participants experienced emotional bond, goal agreement, and task assignment. Findings indicate that AI coaching effectively accelerates goal clarification and provides structured task suggestions, yet it does not generate a coaching presence. In its absence, interactions were described as transactional and emotionally unresponsive; relational engagement weakened, goal agreement was less co-constructed, and task intentions were weakly internalized. Experience level shaped evaluations: coaching-educated and certified participants identified relational deficits early and judged AI more critically, whereas novices initially valued efficiency but later reported a lack of partnership and motivation. These findings highlight a limitation for AI coaching—procedural assistance without relational depth struggles to sustain a transformative working alliance. The study contributes to AI-relevant coaching and offers practical insights for developing emotionally responsive AI coaching platforms, including empathic attunement features, adaptive conversational feedback, and hybrid human-AI models.

## Introduction

Artificial intelligence (AI) has been rapidly applied to fields traditionally relevant to human interaction such as coaching, counselling, and leadership development; however, research on this topic remains largely focused on functional dimensions [1–3]. AI coaching mimics the cognitive and conversational styles of human coaches and researchers have examined whether it can cultivate the relational understanding and emotional engagement important to build coaching relationships [4,5]. Although some scholars argue that current AI coach systems lack the ability to generate the genuine empathy and rapport required to replace human coaches, others report positive resulting in goal-oriented contexts which suggest that AI coaching may function better as a complement than a substitute [6–8].

The working alliance refers to an emotional bond, goal agreement, and task assignment that are imperative for effective coaching practice [9–11]. While emerging research attends to the AI coaching’s functional outcomes, such as goal structuring and feedback quality, a significant gap persists in understanding the client’s lived experience, particularly within the affective and relational dimensions of the working alliance [1,12,13]. Consequently, it remains underexplored whether current AI systems can meet the nuanced relational demands essential for transformative coaching, a role fulfilled by human coaches [1,3,7].

To address this gap, this study answers the following research question: How do client participants experience and interpret the working alliance – emotional bond, goal agreement, and task assignment – along with coaching presence in AI coaching? This overarching question is supported by the following sub-questions:

(1) How do client participants perceive and experience emotional bond, goal agreement, and task assignment with an AI coach?
(2) How does the (absence of) coaching presence shape those experiences?

Adopting Bordin’s [9] working alliance, this study employed reflexive thematic analysis [14] to examine data collected from pre- and post-AI coaching sessions from 15 client participants with diverse coaching backgrounds (e.g., novice, coaching-educated, and certified coaches). Our findings will enable policymakers, Human Resources Leaders, and professional coaches to design AI coaching that better integrates coaching presence and the relational elements essential to successful coaching.

## Literature review

### Defining the working alliance in coaching

**Fig 1:**
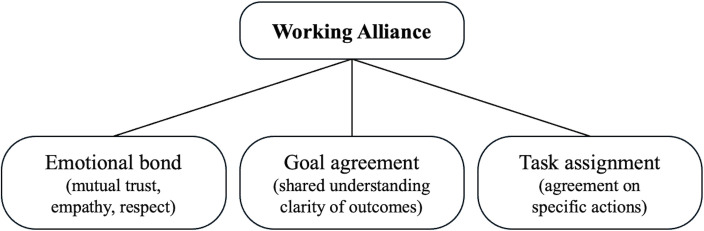
Components of the working alliance.

Coaching is a dialogue that fosters client development, supporting them in achieving their personal and professional goals [15–17]. Effective coaching depends on trust and cooperation within the coach–client relationship [18–20]. We conceptualize this relationship as the working alliance [9], initiated in psychotherapy and then adapted in coaching research [12,21]. The working alliance refers to a collaborative partnership comprising an emotional bond, goal agreement, and task assignment [9,22,23]. These three components (Fig 1) establish psychological safety, intrinsic motivation, and sustained engagement, facilitating deeper self-awareness, meaningful behavioral change, goal pursuit, and performance improvement [10,21].

A working alliance elevates coaching outcomes, including goal attainment, behavioral change, and psychological well-being [21,24]. Coaching effectiveness increases when clients engage in co-constructing goals and selecting meaningful tasks because of the heightened motivation, accountability, and sense of agency fostered through collaborative engagement [25,26]. A recent meta-analysis confirmed a moderate-to-strong positive correlation between the working alliance and coaching effectiveness, demonstrating their central role in various contexts [21]. The underlying reason for this effectiveness is that a robust working alliance creates a secure relational environment, enabling clients to explore personal insights and commit to meaningful behavioral changes [21,25,27].

### AI coaching and limitations of the working alliance

With advancements in machine learning, natural language processing, and personalized recommendation systems, AI coaching has emerged as an alternative to human coaching [5,28]. AI coaches provide coaching to multiple clients and are cost-effective, enhancing accessibility and affordability [29–31].

AI coaching fosters meaningful psychological and behavioral outcomes, such as enhanced self-awareness, increased self-efficacy, reduced stress, meaningful behavioral changes, and goal attainment [3,28]. Participants in AI-based leadership coaching report improvements in goal clarity, stress management, and self-efficacy owing to the structured support and continuous feedback provided by AI coaches [4,5]. However, uncertainty remains regarding whether traditional working alliance constructs – emotional bond, goal agreement, and task assignment – can be adapted to the AI coaching context [32,33].

Scholars criticize the structural limitations of AI coaching on the traditional working alliance constructs. AI lacks the nuanced cognitive and emotional capacities required for interpreting non-verbal communication and subtle emotional shifts that are essential for building genuine emotional bonds [6,34]. Regarding goal agreement, AI coaches often fail in collaborative goal construction during dynamic interactions owing to technical constraints and limited contextual responsiveness [3,5]. In task assignment, AI coaching provides general guidance but remains limited in customization because of predefined algorithms that lack responsiveness to individual client demands [6,29]. These constraints highlight significant relational challenges that AI coaching faces within the working alliance dimensions.

Therefore, AI coaching substitutes procedural aspects but lacks the deeper relational connection and collaboration necessary for effective human coaching [12]. Thus, it should be viewed as a complementary rather than a complete substitute for human coaching [4,35].

### Coaching presence

Coaching presence is recognized as a foundational competence enabling coaches to form effective relationships through mindful, attentive, and responsive interactions [36–38]. It involves remaining conscious, open, flexible, grounded, and engaged, demonstrating empathy, emotional self-regulation, comfort with uncertainty, and the intentional use of silence to foster deeper exploration and insight [38].

Coaching presence is not only a technique but also a holistic way of being, integrating mindful self-awareness, authentic connection, relational attunement, and embodied engagement [36,37]. It facilitates a relational atmosphere that enhances psychological safety, mutual understanding, and meaningful engagement, enabling clients to explore deeper identity, motivation, and goals [39,40].

Despite widespread recognition, researchers encounter challenges in measuring coaching presence because its subjective and relational nature limits the development of validated instruments [37,41]. For instance, a 360-degree feedback, attempted indirect assessment that fails to capture relational nuances, such as emotional attunement and genuine responsiveness, highlights the need for more comprehensive measurement tools [36,37]. However, the coaching literature demonstrates that structured reflective practice, mindfulness training, and supervision can cultivate coaching presence, underscoring its potential for intentional development [37,40].

AI coaching offers procedural clarity but struggles with emotional responsiveness and relational depth, making it crucial to address the role of coaching presence [6,8]. Exploring how relational dimensions such as coaching presence can be integrated into AI systems is essential for guiding the development of adaptive empathic responses.

### Gaps in existing research and the need for this study

The majority of AI coaching research concentrates on survey results and system design which rely on quantitative assessments [3,5,42]; however, how clients interpret the working alliance in personal and relational ways is underexplored [12,23]. Only a small number of studies have investigated coaching presence in AI-mediated contexts, and they often neglect participants’ relational and empathetic responsiveness [3,6].

The majority of research participants are students or experimental samples rather than working professionals [3,28]. Furthermore, little is known about how clients’ previous coaching experience or professional coach training affects their perception of the working alliance in AI-based coaching [21,23,42]. Understanding how different coaching related professional backgrounds shape AI coaching is critical for effective implementation within organizations.

To address the gaps in understanding how prior coaching education and professional certification influence AI coaching, we examined Human Resources Leaders in Korean organizations with varied coaching experience (e.g., novice, coaching‑educated, and certified). The sampling approach aimed to capture diverse experiences of working alliance elements and coaching presence, to shed light on how prior coaching education or professional certification shapes AI-based coaching.

## Theoretical framework

### Interpretive application of the working alliance

Adopting Bordin’s [9] working alliance, we explored clients’ AI coaching experiences through the lens of emotional bond, goal agreement, and task assignment. We adopted Braun and Clarke’s reflexive thematic analysis [14] to understand how participants adapt and construct their meanings through AI interactions. This approach allows us to investigate how the working alliance concept—which originated in psychotherapy and was later adopted in coaching research [21]—translates into relational dynamics within AI coaching.

Central to this framework is coaching presence, defined as intuitive responsiveness, and empathic attunement—because it enables the effective integration of working alliance components [36,43]. It provides a relational foundation that influences client interpretations and experiences of emotional bond, goal agreement, and task assignment within AI coaching.

### Integrated analytical framework of the working alliance

Within this framework, the working alliance constructs, emotional bond, goal agreement, and task assignment, are explored as constructed relational elements rather than static or predetermined characteristics. The emotional bond is examined through client interpretations of relational viability, given AI’s inherent limitations in interpreting nuanced emotional cues that influence perceptions of trust and empathy in AI interactions [44]. Goal agreement is framed to focus on clients’ interpretations of AI’s standardized goal-setting processes and their effectiveness in responding to clients’ evolving objectives and emotional contexts. Here, the focus is on the interpretive tensions caused by AI’s limited flexibility and relational engagement [7,45]. Similarly, task assignment is analyzed through client interpretations regarding AI’s structured yet limited adaptability to individualized contexts. This perspective emphasizes the interpretive gap that clients experience between procedural task suggestions from AI and meaningful relational engagement, that promotes deeper internalization and motivation [3,4].

We conceptualized coaching presence as a foundational relation activated through self-awareness, embodied engagement, empathic attunement, and moment-to-moment responsive co-construction [36,38,39]. Accordingly, coaching presence is different from the three working alliance components: Emotional bond (i.e., experienced trust, safety, and relational connection), goal agreement (i.e., a shared understanding of desired outcomes), and task assignment (i.e., agreement on specific actions) [9]. In our analysis, coaching presence facilitated as a condition that shaped whether participants experienced bond, goal agreement, and task assignment as relationally meaningful, beyond procedural exchanges, in AI coaching. Table 1 outlines the conceptual boundaries and sensitizing analytic prompts that guided our reflexive coding.

**Table 1:** Conceptual boundaries and analytic indicators distinguishing coaching presence from the working alliance components.

Construct	Analytic role in this study	Boundary definition	Generalized analytic indicators used in coding
Coaching presence	Cross-cutting process-level stance shaping the alliance	Moment-to-moment attentiveness, empathic attunement, and responsive co-construction that conditions whether bond, goals, and tasks feel relational (vs. procedural) in AI coaching.	Follows client meaning/context; empathic acknowledgment + affect exploration; adaptive inquiry/reflection; tolerates uncertainty; intentional pause/pacing aligned with client agenda.
Emotional bond	Working alliance component (bond)	Client-experienced trust, psychological safety, and relational connection (i.e., perceived relational quality).	Felt warmth/support; willingness to disclose; feeling understood/validated; continuity/reliability across turns.
Goal agreement	Working alliance component (goals)	Co-constructed alignment on desired outcomes and success criteria (beyond goal clarity alone).	Goals in client’s terms; explores why it matters (meaning/value); alignment checks/renegotiation; success criteria; goals refined as dialogue unfolds.
Task assignment	Working alliance component (tasks)	Agreement on actions that advance goals, including feasibility and client ownership/commitment.	Specific action steps; feasibility/prioritization; barriers/resources; ownership/commitment language; light accountability planning (as applicable).

## Materials and methods

This exploratory qualitative inquiry employed reflexive thematic analysis to examine how Human Resources leaders experienced the AI coaching working alliance and to clarify the role of coaching presence as a context-sensitive and emergent construct. To investigate this, we conducted paired pre- and post-interviews with 15 senior Human Resources Leaders, supported by session artifacts. We purposefully sampled across three expertise groups (novice, coaching-educated and certified) to compare perspectives on bond, goal and task. Trustworthiness was established through triangulation, regular meeting, peer debriefings, a reflexive journal, and an audit trail.

### Research design

We applied the reflexive thematic analysis [14,46] to examine how Human Resources leaders experienced and interpreted the working alliance in AI coaching. This approach allows us to thematically understand how client participants derive meaning from their AI coaching experiences. The analysis followed six phases: Data familiarization, generating initial codes, constructing and reviewing themes, refining and naming themes, and describing findings [14,47]. We focused on how participants experienced the emotional bond, goal agreement, and task assignment during AI coaching.

### Participants

We adopted purposeful sampling, network sampling [48], to recruit 15 client participants (seven men and eight women) from major IT companies in the Republic of Korea. All client participants had at least 15 years of experience in Human Resources management and talent development and held leadership positions. To explore perceived differences in the working alliance, we categorized participants into three groups based on their coaching expertise:

Group 1: Novice client participants (n = 5) had no prior coaching experience.

Group 2: Coaching-educated client participants (n = 5) completed formal coaching education programs (e.g., through the Korea Coach Association or International Coaching Federation without certification).

Group 3: Certified coaches as client participants (n = 5) completed educational and formal certifications (e.g., Korea Professional Coach and Professional Certified Coach) from recognized institutions.

The coaching expertise criteria were based on recognized coaching education programs and professional certifications from the Korea Coach Association and International Coaching Federation. All client participants voluntarily consented and were informed about the research objectives, procedures, and ethical considerations. Initially, 18 participants agreed to participate; however, owing to scheduling conflicts, three withdrew, leaving 15 participants. Participant recruitment for this study occurred from November 1, 2024, to December 31, 2024. The data collection process entailed three preliminary interviews, pre-/post interviews, coaching sessions, and member-checks, which occurred between January 13, 2025, and April 6, 2025. Table 2 presents the demographic details of these participants.

**Table 2:** Participants’ demographic characteristics.

Group	Pseudonym	Gender	Education	Industry	Job Title	Years of Experience	Experience of Coaching Education	Certification of Coaching
G1	Jin	M	Bachelor’s degree	IT (Platform)	Team Leader	18	None	None
	Min	F	Bachelor’s degree	IT (Messenger)	Director	23	None	None
	Jun	M	Bachelor’s degree	IT (Game)	Director	27	None	None
	Soo	F	Bachelor’s degree	IT (Messenger)	Team Leader	20	None	None
	Ara	F	Bachelor’s degree	IT (Game)	Team Leader	18	None	None
G2	Hana	F	PhD (in progress)	IT (Platform)	Team Leader	24	100 hours	None
	Jina	F	Bachelor’s degree	IT (Game)	Director	16	40 hours	None
	Yuri	F	Master’s degree	IT (Platform)	Director	22	20 hours	None
	Hoon	M	Bachelor’s degree	IT (Platform)	Director	27	20 hours	None
	Minho	M	Bachelor’s degree	IT (UI/UX)	Director	20	40 hours	None
G3	Mina	F	Bachelor’s degree	IT (Messenger)	Team Leader	15	100 hours	KPC^a^ by KCA^b^
	Jae	M	Master’s degree	IT (Messenger)	Director	23	70 hours	KAC^c^ by KCA
	Somi	F	Master’s degree	IT (Messenger)	Team Leader	23	150 hours	KPC by KCA, PCC^d^ by ICF^e^
	Joon	M	Master’s degree	IT (Game)	Director	17	150 hours	KPC by KCA
	Nara	F	PhD (in progress)	IT (Game)	Team Leader	20	130 hours	KPC by KCA

^a^Korea Professional Coach.

^b^Korea Coach Association

^c^Korea Associate Coach

^d^Professional Certified Coach

^e^International Coaching Federation

### Data collection procedure

#### Interview design, content, and transcription.

To understand client participants’ experiences, the first author conducted semi-structured interviews with 15 client participants twice, before and after their AI coaching experiences. The first author maintained a neutral ‘not knowing posture’ [49] throughout interviews to avoid influencing client participants’ responses [50,51]. The client participants described their expectations, concerns, and perceived differences between AI and human coaching during pre-interviews. All client participants shared experiences related to the working alliance, their overall impressions of AI coaching, differences from human coaching, and potential future applications during post-interviews. New exploratory questions emerged as the authors engaged with the participants and reviewed transcripts [52]. All interviews were conducted online (Zoom or phone), with pre- and post-interviews averaging 30 minutes and 50 minutes, respectively, and were transcribed with an AI-based transcription tool (Clova Note). The client participants provided supplementary materials, such as screen captures of each session and audio recordings, to contextualize their participation. We corrected transcription errors by comparing the transcripts with original recordings. We recorded 1,123 minutes of interviews that were transcribed into 725 pages.

#### AI coaching experience.

**Fig 2:**
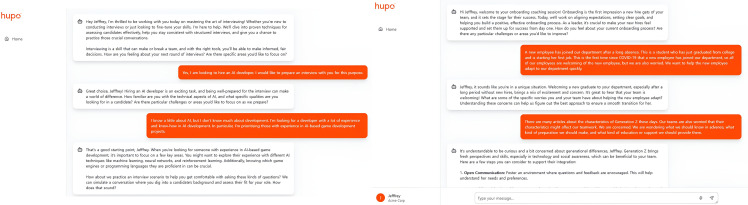
Hupo AI coaching dialogue interface (screenshot from a researcher-run test session).

Client participants engaged in AI coaching on the Hupo platform (Singapore), which enables voice- and text-based dialogue between a client and an AI coach. Using a standardized protocol, each client participant selected one of three scenarios—new employee onboarding, career development, or job interview—chosen to reflect common HR-related coaching topics and to support the participants’ consistent experiences. Each client participant completed one 40-minute session in a location of their choice. After the session, client participants provided screen captures or voice recordings, which we used in the post-interview to support recall, interpret client participants’ interpretations, and verify accuracy. Scenario selection by client participant group is reported in S1 Table. We compared coded patterns across scenario types as a sensitivity check; the thematic structure remained consistent. Fig 2 presents a screenshot of the Hupo interface captured during a researcher-run test session under the same platform configuration as the study sessions and contains only test data.

### Data analysis

We employed Braun and Clarke’s [14] reflexive thematic analysis to explore client participants’ experiences and meaning negotiation. The first author, a certified coach, brought a heightened sensitivity to the relational nuance of coaching, which likely influenced the identification of coaching presence as a major theme. The second author provided ongoing debriefing sessions to ensure analytical transparency and explore alternative perspectives to better catch relational dimensions [53,54].

First, we familiarized ourselves in all pre- and post-interview transcripts and session artifacts through repeated reading and analytic memo-writing, attending to client participants’ accounts in relation to the three working alliance components [14,46]. Treating these components as sensitizing concepts, we generated 100 initial codes across the dataset and clustered related codes into candidate themes. We refined candidate themes through iterative review of coded extracts and the full dataset, consolidating overlapping patterns and reducing redundancy. This iterative work highlighted coaching presence as a condition shaping how client participants perceived emotional bond, goal agreement, and task assignment in AI coaching. We then finalized a thematic structure comprising four themes (emotional bond, goal agreement, task assignment, and coaching presence) and defined 12 subthemes through reflexive discussion and author debriefing. We wrote up the analysis by weaving an analytic narrative with representative quotations capturing both semantic content and latent meaning [55]; extended code summaries are provided in S2 Table.

### Ethical considerations

This interview-based qualitative study involved minimal risk. aSSIST University, Seoul, Republic of Korea, does not operate an Institutional Review Board for minimal-risk qualitative research; therefore, formal ethical approval was not required under institutional policy.

All client participants received an information sheet describing the study purpose, procedures, confidentiality, and data protection, and provided written informed consent prior to participation [14,56]. Client participants could withdraw at any time. We collected interview recordings and AI coaching session materials only after consent and used the data for the stated research objectives. All records were de-identified prior to analysis and reporting by removing personal and organizational identifiers and using pseudonyms [57,58]. De-identified data were stored on password-protected systems, accessible only to the authors.

We followed Lincoln and Guba’s [59] framework to ensure the study’s trustworthiness. Methodological credibility was established via peer debriefing with the second author and monthly meetings with colleagues; and thick descriptions, supported by a detailed audit trail. The first author’s reflexive journal further ensured analytical transparency and rigor throughout the research process.

## Findings

**Fig 3:**
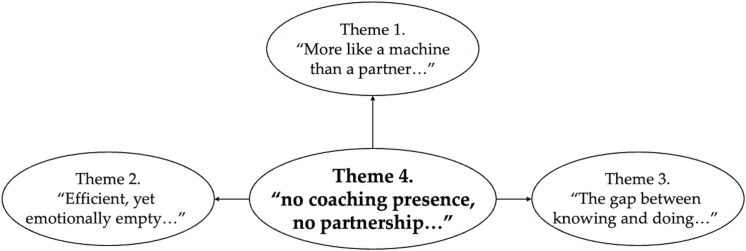
Thematic structure linking coaching presence and working alliance.

Our analysis revealed four primary themes that captured client participants’ experiences of AI coaching. These themes reflected the structural components of the traditional working alliance and the absence of a coaching presence. This absence operated as the central organizing concept, shaping how client participants made sense of an emotional bond, goal agreement, and task assignment in their AI coaching. The structure derived from the reflexive thematic analysis [14,46] not only shows the perception of these relational components but also how they were reconfigured in the absence of human relational responsiveness [60,61]. Fig 3 and Table 3 summarize the relationships among these themes, their sub-elements, and representative quotations from the client participants.

**Table 3:** Summary of key themes, sub-themes, and illustrative client participant quotes.

Theme	Sub-Themes	Illustrative Quotes	Pseudonym	pre/post
“More like a machine than a partner…”	Lack of basic trust in AI coaching Recognition of AI coach as non-relational Acceptance of AI coach as non-human	Since the AI coach lacks any background knowledge about me and is essentially a machine, I felt I could speak freely and comfortably about whatever I wanted	G1, Ara	pre
		Because I could not establish trust in the AI coach, I found myself reluctant to share deeply. Consequently, the responses became increasingly dry and impersonal	G2, Hoon	post
		Even when the AI responded diligently, the interactions still felt mechanical, partly due to its unrefined and insufficiently sampled voice. Once I realized clearly that I was interacting with a machine, my initial expectations diminished significantly. I resigned myself to accepting this mechanical tone, recognizing that it would persist unchanged throughout the entire session, starting from rapport building	G3, Joon	post
“Efficient, yet emotionally empty…”	Fast but unfeeling goal setting Structured but emotionally unresponsive Lack of empathy, care, and co-agency	Given the AI’s extensive data-processing capabilities, I expected rapid responses and efficient interactions	G1, Min	pre
		Empathy is foundational to effective coaching; however, I doubted the AI could adequately grasp the emotional context underlying our conversations	G2, Yuri	post
		Although the AI coach provided accurate and relevant responses, the lack of emotional empathy meant that its goal-setting process lacked motivational direction and depth	G3, Mina	post
“The gap between knowing and doing…”	Informationally rich but affectively unresponsive One-time advice without motivational push Lack of emotional investment hinders action	Ultimately, I did not feel a sense of genuine resolution or accomplishment	G1, Min	post
		The interaction proceeded rapidly, but because each exchange ended abruptly without further engagement, I did not feel personally compelled or motivated to take action	G2, Hoon	post
		With human coaches, conversations naturally foster a sense of shared responsibility and motivation toward taking action. Conversely, interactions with the AI coach lacked this interactive component, significantly reducing my internal motivation	G3, Nara	post
“No coaching presence, no partnership…”	Absence of coaching presence No sense of being accompanied Working alliance weakened	Due to the absence of empathy and reliance solely on informational exchange, I perceived little meaningful interaction or relational connection with the AI coach	G1, Soo	post
		Human coaches are often selected based on their professional profiles, allowing for mutual respect to develop during interactions. However, I held no such expectations for the AI coach	G2, Hana	pre
		Although our conversation technically progressed, I never genuinely felt connected, supported, or empathically understood by the AI	G3, Somi	post

In Fig 3, Theme 4 (no coaching presence, no partnership...) operated as a structuring condition that connected the three working alliance components and reflected Bordin’s conceptual framework.

### AI coach’s limited communication capabilities: “More like a machine than a partner…”

In verbal and non-verbal communications, client participants described AI as a mechanical tool rather than a relational partner, indicating an insufficient emotional bond owing to limited human language. Although some participants noted the strength of AI coaches’ linguistic competency and prompt response, they emphasized that functional linguistic strengths did not translate into emotional engagement or offer sufficient psychological safety expected from a coaching relationship. The participants considered AI coaching as an information-processing mechanism without empathy or trust:

When I tried AI coaching, the voice sounded very mechanical and stiff. While it was possible to have a conversation, I didn’t feel like it was empathetic nor connected to me. When I shared something deep, it felt like it was just reciting a prepared response, which made me feel uncomfortable. I couldn’t feel any warmth or trust that comes from a relationship; it just felt like a cold, dry tool. (G3, Joon, post)

Even before experiencing AI coaching, one client participant indicated a preconception about the emotional limitations of AI coaching:

The greatest comfort in coaching comes from the coach’s facial expressions and body language. It is important to feel that they understand you. I doubt that AI can do that. Suppose there is non-verbal communication, such as facial expressions or body language, and only words are exchanged. In that case, I am not sure if that alone is enough to provide sufficient empathy or comfort. I don’t think it can replace the warmth that humans provide. (G1, Soo, pre)

These accounts reveal that the client participants did not expect or experience emotional bonding through AI because of restricted non-verbal communication. The client participants with prior coaching education or certification (Groups 2 and 3) demonstrated a more explicit and critical awareness of AI’s emotional limitations. They expressed that, unlike human coaches, the AI coach had insufficient communication capabilities to build an emotional bond, linking to the central organizing concept – the absence of a coaching presence.

### AI coach’s superficial goal-setting approach: “Efficient, yet emotionally empty…”

Although the AI coach suggested clear and structured goals, the client participants recognized that it was limited in creating meaningful goal agreement. Such agreement requires mutual understanding, relational depth, and shared meaning-making between coach and participant. Human coaches guide clients in developing goals through mutual understanding and contextual empathy [22,25]. The AI coach was executed executing a pre-scripted procedure that delivered answers rather than fostering a meaningful goal agreement. The client participants acknowledged the AI coach’s speed and clarity but expressed dissatisfaction with its inability to respond to emotions or the depth of objectives:

When setting goals, the AI was quick in processing information and suggesting goals, which was impressive. But, you know, goal-setting isn’t something that can be decided just based on information, right? It requires discussing, sharing, and understanding how I feel, what’s going on in my life, and working things out together. The AI didn’t create any emotional connection or understanding in this aspect. Honestly, it felt like it was just following a preset manual, skipping the detailed and personal parts that matter. That was pretty disappointing. (G2, Hoon, post)

The relational feedback, discussing, sharing, and understanding is important to help client participants explore and find their own meaningful goals. Often, client participants do not know their goals and meaningful purposes; therefore, they need dialogic engagement to expand and deepen the meaning. During the pre-interview, one client participant noted that engagement is like taking a journey together:

With a human coach, I feel like we’re working together to set goals. I’m worried about how much an AI coach can make me feel like it’s ‘on the same journey’ during that process. (G2, Yuri, pre)

The apprehension of this client participant stems from a fundamental belief that goal agreement is relational and collaborative. Effective goal setting requires not only a structured direction but also qualities that are absent in the AI context, such as emotional investment, shared understanding, and mutual support [62,63]. The client participants viewed the AI coach as a reactive tool rather than a co-constructive partner. Although the goal-setting procedure was complete, it left participants feeling disconnected. This experience revealed their implicit view that genuine goal agreement is an interpretive act, built on the relational sensitivity and emotional engagement the AI lacked. Accordingly, the AI coach failed to support the participants in achieving a meaningful goal agreement.

Prior coaching experience shaped the participants’ interpretations of AI coaching’s goal setting and relational limitations. The client participants with formal education in coaching (Groups 2 and 3) expressed dissatisfaction with AI’s superficial relational approach, which was sensitive to the absence of a genuine coaching presence. Conversely, the client participants without prior coaching experience (Group 1) focused on procedural efficiency and recognized relational limitations later. These differences underscore the critical influence of prior experience on expectations and evaluations of goal-setting interactions within AI coaching, reflecting the central organizing concept – the absence of a coaching presence (Theme 4).

### AI coach’s motivational limitations in task assignment: “The gap between knowing and doing…”

The client participants acknowledged the structured clarity of the task assignments. However, they described a critical gap between receiving tasks and developing the genuine motivation to act upon them. This reveals that the task assignment through AI coaching lacks a key component: the ability to foster genuine motivation. The effectiveness of a task assignment relied more on motivation, internalization, and relational support than on informational clarity. The successful implementation of tasks involves trust, shared responsibility, and emotional alignment – elements fostered by a human coach’s relational presence. By contrast, the participants described the AI coach as delivering tasks without stimulating the psychological activation necessary for meaningful follow-through:

I heard a lot of good advice; however, I felt unmotivated to act on it. It was a decision I made after thinking and immersing myself in it; it felt like someone else had organized it and just handed it to me, so I couldn’t make it my own. (G3, Joon, post)

Furthermore, the client participants emphasized that effective task assignment requires more than clear informational exchanges, necessitating active emotional acknowledgment and supportive relational dialogue. Active emotional acknowledgment was critical, as it contributed to fostering participants’ intrinsic motivation and commitment towards task implementation. In AI coaching interactions, the client participants often started without defined goals or motivational clarity, highlighting a significant contrast to the emotional and relational support inherent in human coaching. Human coaches engage clients through empathetic listening, relational dialogue, and emotional encouragement, influencing their motivational readiness. Before experiencing AI coaching, participants expressed concerns about the potential lack of motivational and emotional elements present in human coaching:

I think an AI coach would give me guidance right away. But I’m not sure if I’d feel like; I want to do it’ because, in coaching, it’s not just about receiving clear instructions. Usually, the coach listens to my situation closely, acknowledges my feelings, and helps me feel motivated or committed. With an AI coach, I wonder if that motivational push and emotional connection would be there. (G2, Jina, pre)

These statements emphasize the client participants’ underlying belief that effective task assignment during coaching interactions requires not only cognitive guidance but also relational depth, affective engagement, and shared meaning-making. The participants perceived the AI coach as an information provider, lacking the relational depth necessary to function as a genuine co-constructive partner. This relational deficit weakened participants’ capacity to translate knowledge into meaningful actions. Such relational disconnection, driven by the absence of a coaching presence, is the primary reason for reduced motivational activation and task implementation effectiveness in AI coaching.

Furthermore, the participants’ previous experiences with human coaching influenced their assessments of AI coaching in terms of relational depth and motivational capacity. The participants with formal coaching or certification (Groups 2 and 3) were dissatisfied with the superficial relational approach of the AI coach, citing its inability to facilitate genuine emotional engagement, accountability, and internal motivation. Conversely, those without prior coaching experience (Group 1) emphasized procedural efficiency and clarity, effectuating a delayed recognition of the relational and motivational limitations inherent in AI coaching. These variations illustrate the significant role of prior experience in forming client participants’ expectations, perceptions, and evaluations regarding the motivational effectiveness of task assignments in the AI coaching context.

### AI coach’s relational deficit: “No coaching presence, no partnership…”

The client participants identified the absence of a coaching presence as the most critical limitation in their experiences with AI coaching, undermining meaningful and sustainable interactions. Despite the procedural clarity and structured support provided by the AI coach, the participants described their interactions as unresponsive, disconnected, and unfulfilling. The client participants described coaching presence, marked by deep relational attunement, authentic responsiveness, and intuitive engagement, as essential for building meaningful interactions and sustainable coaching alliances. However, these elements were absent from their interactions with the AI coach. Accordingly, the client participants characterized their AI coaching experiences as transactional, unresponsive, and unfulfilling, relational deficits compared with their human coaching experiences:

It didn’t feel like it understood my story and was with me. Its responses felt more like echoes than real empathy. I had to take control and initiate every step. It didn’t feel like having a coach there. (G1, Min, post)

It felt more like I was cherry-picking what I wanted to know rather than having a meaningful dialogue. The empathy felt perfunctory, and I questioned whether it resonated with me. My questions were answered, but it lacked a human touch. It felt as if I were responsible for driving the conversation without genuine partnership. (G2, Mina, post)

These client participants’ accounts highlight the significant gap between AI’s procedural competence and relational depth, which is essential for effective coaching. Even before experiencing AI coaching, client participants expressed skepticism regarding the AI coach’s ability to substitute for coaching presence:

Human coaches are warm, empathetic, and intuitively attentive beyond spoken words. That’s the type of presence I value and expect. I’m doubtful an AI coach can empathize or offer that sense of presence. I see this as the biggest distinction between AI and human coaching. (G2, Yuri, pre)

Post-session experiences confirmed these concerns by reinforcing the participants’ perceptions of interactions as transactional rather than relational:

The conversation didn’t deepen or progress. It felt as if the AI coach was responding to each isolated input. There was no real progression, and more importantly, no genuine sense that someone was with me. (G3, Jae, post)

These experiences show that the client participants’ expectations were unmet owing to the inherent relational limitations of current AI systems, even though these systems have procedural strengths such as consistency and efficiency. Thus, the absence of coaching presence, a core relational quality, explains the participants’ inability to form emotional bonds (Theme 1), achieve meaningful goal agreements (Theme 2), and commit to assigned tasks (Theme 3).

The client participants’ prior experiences with human coaching influenced how they perceived the relational limitations of AI coaching. Specifically, the client participants with coaching education and certification (Groups 2 and 3) articulated more explicit dissatisfaction with the superficial relational approach of the AI coach, emphasizing the absence of authentic emotional engagement, empathic understanding, and intuitive responsiveness characteristic of an effective coaching presence. Their expectations were shaped by rich human coaching experiences, highlighting stark contrasts with AI-mediated interactions. Conversely, the client participants without coaching experience (Group 1) emphasized procedural clarity and efficiency but recognized relational shortcomings. This variance demonstrates how prior coaching exposure affects client expectations, perceptions, and evaluations regarding relational dynamics in AI coaching.

These findings underscore that without coaching presence, the relational foundation essential for a transformative coaching alliance collapses, leaving AI coaching interactions transactional. The absence of a coaching presence is a fundamental barrier limiting the motivational, relational, and emotional effectiveness of AI coaching in organizational contexts.

**Fig 4:**
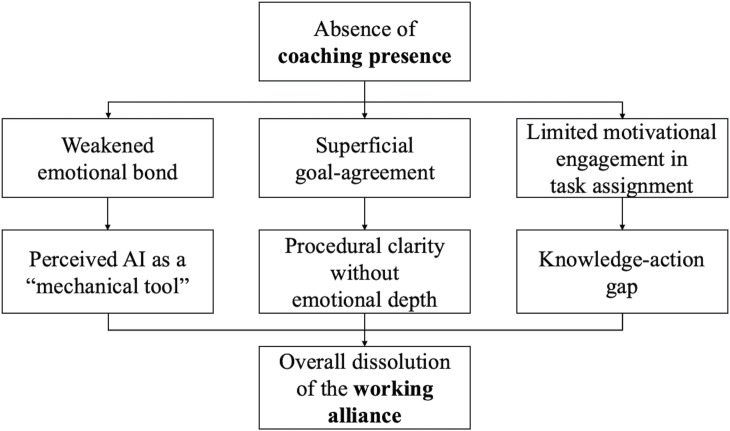
Impact of absent presence on working alliance components.

Fig 4 visualizes this integrative conceptual framework and illustrates how a lack of a coaching presence disrupts relational coherence among working alliance components in the AI coaching context.

## Discussion

Our findings show that AI coaching supports procedural aspects of the working alliance but constrains its overall development in organizational settings. Across the three alliance components—emotional bond, goal agreement, and task assignment—AI coaching provided structure and consistency, yet client participants experienced limited coaching presence. Participants described coaching presence as the relational condition through which bond, goals, and tasks were experienced as meaningful alliance elements rather than as efficient, outcome-focused outputs.

First, AI coaching demonstrates a critical divergence between procedural efficiency and relational viability in the working alliance [4,5,8]. In our analysis, participants experienced this strength as efficient goal structuring and information-dense task support. Meanwhile, they described an absence of being accompanied—an experiential marker of coaching presence grounded in mindful, attentive, and responsive interaction [36–38]—which they linked to weaker trust and psychological safety in coaching relationships [10,18–20]. These findings suggest that procedural efficiency supports clarity, whereas coaching presence supports relational viability.

Second, AI coaching produces goal clarity and task structure but fails to foster the goal agreement and motivational internalization that emerge through dialogic co-construction. Goal agreement requires shared meaning and mutual commitment developed through dialogue [9,22,23]. Participants described AI coaching as producing procedural clarity without emotional depth, leaving goals organized but less owned. A similar mechanism appeared in task assignment. AI coaching supported action planning, yet participants reported weaker internalization and reduced motivational engagement when responses lacked attuned responsiveness. This pattern aligns with evidence linking alliance quality to motivation, accountability, and coaching outcomes [21,25,27].

Third, prior coaching experience shaped participants’ capacity to recognize and articulate AI coaching’s relational limitations, distinguishing immediate expert detection from novices’ emergent experiential awareness. Coaching-educated and certified client participants evaluated the AI coach against expectations of empathic attunement and responsive co-construction and identified relational deficits, consistent with evidence linking coach development to sensitivity to presence and relational nuance [37,39,43]. Novice client participants valued AI coaching’s structure and consistency but later reported a weaker sense of partnership and reduced motivational pull. This pattern extends AI-coaching research by specifying how prior coaching exposure and professional coach training shape what clients treat as evidence of alliance quality in AI-based coaching [21,23,42].

This study extends AI coaching research beyond survey-based outcome measures and platform evaluations. Using reflexive thematic analysis of paired pre- and post-session interviews with HR leaders, we examined how participants constructed alliance quality in organizational AI coaching and how they assessed coaching presence across bond, goals, and tasks. This analytic approach identified experiential indicators of coaching presence (e.g., a sense of being accompanied) and demonstrated how prior coaching experience shaped the criteria participants used to evaluate alliance quality.

These findings clarify why the AI coaching literature reports mixed alliance outcomes. Effectiveness depends not on the AI label but on whether interactional cues are enacted in ways clients experience as coaching presence. Barger’s [12] Wizard-of-Oz study reported high working alliance ratings and no significant difference between simulated AI and human coaching after a single session [12], a setting in which expert human coaches delivered the interaction behind the interface. In contrast, our participants engaged with a current AI system situated in organizational coaching scenarios and evaluated the interaction against workplace coaching expectations, which foregrounded the absence of coaching presence. The divergence indicates that AI coaching is not a unitary condition; alliance formation depends on whether cues of attentiveness, responsiveness, and empathic attunement are enacted in ways clients experience as coaching presence, beyond the interface label itself.

### Implications for theory and theory development

We conceptualize the working alliance in AI coaching as conditioned by coaching presence. Prior AI‑coaching literature emphasizes functional capabilities (e.g., goal structuring, task suggestions, automated feedback) and offers a thinner account of how relational and emotional processes operate in AI‑mediated coaching [3–5,28]. Building on Bordin’s [9] model and coaching-presence scholarship [36,37,39], we position coaching presence as the central organizing concept through which emotional bond, goal agreement, and task assignment function as alliance components in AI coaching.

We defined coaching presence as a cross-cutting process stance—mindful self-awareness, embodied engagement, empathic attunement, and responsive co-construction—that operates across alliance components rather than residing only within the bond [36,37,39,40]. This definition strengthens construct boundaries by treating presence as foundational rather than as a bond subdimension. It also distinguishes goal clarity from goal agreement and task assignment from task engagement, explaining why procedural support strengthens clarity whereas coaching presence supports co-construction and commitment [9,22,23,25,26]. In this account, procedural support provides clarity, and coaching presence provides relational depth.

These findings extend working alliance theory to organizational talent development settings and frame alliance quality as a relational mechanism linking AI coaching interactions to motivation and sustained goal pursuit [2,3,21,25–27]. The group pattern suggests that prior coaching exposure shapes what clients treat as evidence of presence and alliance in AI coaching [21,23,42]. Future research can test coaching background and relational expectations as boundary conditions of alliance development and outcomes and can develop measures that capture the moment-to-moment dynamics of coaching presence [37,41].

### Implications for business and management practice

These findings support a complementary coaching design for HR-led people and talent development. AI coaching provides procedural structure and immediacy, whereas human coaching sustains coaching presence [4,36–40,43]. HR can assign AI coaching to structured goal review and task planning and reserve human coaching for conversations in which coaching presence shapes progress.

Implementation should align expectations with user experience. Coaching-educated and certified participants evaluated AI coaching more critically than novice participants, indicating that prior coaching exposure can guide onboarding and communication [21,23,42]. HR can position AI coaching as an on-demand, procedure-oriented resource and clarify referral pathways to human or hybrid support to reduce expectation gaps [3,7].

Programs can allocate coaching modalities based on topic demands. Human coaching suits emotionally salient concerns (e.g., stress, burnout risk, sustained motivation), whereas AI coaching suits brief, repeatable micro-interventions (e.g., rapid goal reviews, structured reflection prompts, simulation-based rehearsal) [29–31]. Hybrid models can support increasingly self-directed client change and growth by combining human sessions that sustain coaching presence with between-session AI support that reinforces task follow-through, shared accountability, and rehearsal of challenging conversations.

## Conclusion

We sought to answer two questions. First, how do client participants perceive and experience the working alliance—emotional bond, goal agreement and task assignment—in AI coaching? We find that AI coaching offers procedural clarity but limited relational depth: Emotional bonds weakly internalized, goal agreement is less co‑constructed, and task assignment is weakly internalized. Second, how does coaching presence shape these experiences? Our findings identify coaching presence as the foundation of the alliance; its absence constrains emotional connection, sustained engagement and goal pursuit, pointing to design features and hybrid human–AI models that center coaching presence to achieve genuine relational effectiveness.

This study has some limitations that provide directions for future research. First, the client participants comprised South Korean IT Human Resources Leaders, limiting the generalizability to other cultural and industrial contexts. Second, the AI coaching experience had structural limitations. It was restricted to single sessions covering three pre‑determined topics and thus did not fully reflect broader coaching needs or realistic scenarios faced by the client participants. Future research could explore how cultural dimensions, such as communication styles or power distance, mediate the perception of AI’s relational deficits. Longitudinal studies tracking multi-session, open-topic engagements are also needed to determine if familiarity can reduce the initial lack of presence.

In conclusion, this study identified coaching presence as a fundamental relational dimension underpinning the working alliance’s effectiveness in AI coaching. AI coaching substitutes procedural components, yet it is constrained by relational limitations in fostering emotional engagement, sustained engagement, and goal pursuit. By advancing a framework that centers coaching presence, our findings offer clear guidance for developing the next generation of AI coaching systems—ones that move beyond procedural efficiency toward genuine relational effectiveness.

## Supporting information

S1 Table Scenario choice of each group. (XLSX)

S2 Table Data_themes and codes. (XLSX)
